# Epicardial adipose tissue is a predictor of ascending aortic dilatation in hypertensive patients, but not paracardial adipose tissue

**DOI:** 10.1186/s12872-020-01431-2

**Published:** 2020-03-19

**Authors:** Onur Argan, Eyup Avci, Tarik Yildirim, Ozgen Safak

**Affiliations:** Department of Cardiology, Balikesir University Medical Faculty, 10440 Balıkesir, Turkey

**Keywords:** Epicardial adipose tissue, Ascending aortic dilatation, Hypertension

## Abstract

**Background:**

Ascending aortic aneurysms are one of the major causes of mortality. In recent years, there is a growing interest of epicardial adipose tissue (EAT) and related diseases. The aim of this study was to investigate the relationship of EAT, and PAT with ascending aortic dilatation (AAD).

**Methods:**

We included 934 patients with hypertension in this study**.** The patients were evaluated by a complete transthoracic echocardiographic examination, including measurements of EAT, PAT, and aortic dimensions. Aortic size index (ASI) was used for diagnosing AAD. The patients were divided into two groups: dilated ascending aorta diameter (ASI ≥ 21 mm / m^2^, *n* = 102) or normal aortic diameter (ASI < 21 mm / m2, *n* = 832) according to the ASI. Characteristics of these patients were compared before and after propensity score matching analysis.

**Results:**

Patients with AAD were older (72.3 ± 11.6 vs. 61.7 ± 12.7 years, *p* <  0.001), had more female gender (66% vs. 54%,*p* = 0.021) than patients with normal ascending aorta (AA). After propensity score matching analysis (77 vs*.* 77), EAT [OR:1.461, %95CI (1.082–1.974), *p* = 0.013] was independently associated with AAD in multivariate logistic regression analysis. In ROC curve analysis, EAT > 0.45 cm had 51.9% sensitivity and 62.3% specificity [AUC = 0.617, *P* = 0.012, 95% CI (0.529–0.707)].

**Conclusion:**

Based on our findings, increased EAT may be suggested as an independent risk factor for AAD due to local or systemic effects in hypertensive patients.

## Background

Ascending aortic aneurysms and dissections are one of the major causes of mortality [1]. Despite important recent development in understanding of its pathogenesis, the role of haemodynamics and other reasons are partially known yet. The pathogenesis of ascending aortic dilatation (AAD) includes several factors with systemic and local effects. The aetiology is multifactorial, including environmental and genetic factors that play important roles in progression of aortic disease [2]. Given that the pathogenesis of AAD maintains unclear, the main reason seems to be related to the deficiencies or defects in structural proteins, such as collagen and elastin in the aortic wall [3]. Changes of the extracellular matrix are suggested as a key factor in the pathogenesis of AAD [2–4]. Cystic medial degeneration responsible for weakened aortic wall is related to the degeneration of smooth muscle cell and elastic fibre apoptosis [5]. Also, the inflammatory process associated with atherosclerosis causes aortic dilatation [6].

Hypertension is one of the considerable risk factors for AAD. AAD is often observed in hypertensive patients compared with normotensive individuals and is correlated with cardiac and noncardiac organ injury in hypertensive patients [2–4]. Hypertension is one of the most common diseases and a worldwide health problem. It is related to stroke, renal disease, cardiac disease, aortic disease, mortality and morbidity. It generally progresses silently until it causes end organ damage. However, it is a treatable and preventable disease.

In recent years, there is a growing interest towards the effects of epicardial adipose tissue (EAT) on hypertension and cardiovascular risk factors. EAT is a visceral adipose tissue found between the pericardium and the myocardium [7]. It is generally placed in the atrioventricular and interventricular groove throughout the coronary arteries. There is no any fascia between the myocardium and EAT; therefore, they have same microcirculation. EAT secretes antiinflammatory and antiatherogenic mediators and supplies energy to the myocardium [8–10]. However, under pathological conditions, EAT appears to play a critical role in atherosclerosis, hypertension and progression of metabolic diseases as it acts as a proinflammatory and prothrombotic organ [11]. Tumour necrosis factor alpha, interleukin-6 and monocyte chemoattractant protein-1, secreted from the EAT [12], immediately affect atherosclerosis [13, 14]. As a result, increased EAT is related to atherosclerosis [15]. EAT may play a provocative role for AAD with paracrine and systemic endocrine effects on vessels [16].

Paracardial adipose tissue (PAT) is an ectopic fat depot surrounding the heart [17, 18]. It is placed anterior to the EAT and outside the parietal layer of the pericardium [19]. The pericardium restricts the communication between the PAT and EAT. Studies about the different roles between EAT and PAT are limited. A few studies showed that PAT was more closely related with abdominal visceral adipose tissue and metabolic risk than EAT. It is also recommended that PAT might be a marker for increased metabolic risk and visceral adipose tissue on thoracic imaging [17–21].

The focus of the studies has been commonly on EAT, neglecting the potentially additive effects by the PAT on cardiovascular diseases. The aim of this study was to investigate the associations of EAT, and PAT with AAD in hypertensive patients.

## Methods

The present study included 934 hypertensive patients who underwent complete transthoracic echocardiographic examination involving measurements of aortic dimensions, EAT and PAT. When diagnosing AAD, we used the body surface area (BSA)-adjusted classifications. For this purpose, we used Roman’s classification (aortic size index, ASI) [22]. ASI was calculated by the following formula: ASI = AA diameter (mm)/ BSA (m^2^) [23]. AAD was defined as a ASI ≥ 21 mm/m^2^ according to the Roman’s classification [23]. A total of 102 hypertensive patients with AAD were compared with 832 hypertensive patients with normal aortic diameter. Exclusion criteria were bicuspid and rheumatic aortic valve disease, Marfan syndrome, Ehlers-Danlos syndrome, Familial Thoracic Aortic Aneurysm Syndrome, Turner Syndrome and other connective tissue disorders, infectious conditions, restrictive and hypertrophic cardiomyopathy, renal failure requiring dialysis, malignancy and pregnancy. Hypertension was defined as the documentation of a blood pressure of more than 140/90 mmHg or the use of antihypertensive drugs.

The study was approved by the Institutional Ethics Committee and conducted in accordance with the principles set out in the Declaration of Helsinki.

The demographic data included gender, age, body mass index (BMI), a history of coronary artery disease (CAD) and diabetes mellitus (DM). BMI was calculated by the following formula: BMI = weight (kg) / height^2^ (m). BSA was calculated using formule; BSA (m^2^) = ([Height (cm) x Weight (kg)]/ 3600)½ . The echocardiographic data involved EAT, PAT, ejection fraction (EF), left ventricle end diastolic diameter (LVEDD), left atrium (LA) diameter, right ventricle diameter and mitral, aortic and tricuspid valve diseases.

For biochemical data, we collected haemoglobin (Hb), haematocrit (Htc), white blood cell count, platelet, creatinine, urea, HbA1c, aspartate transaminase (AST), alanine transaminase (ALT), total cholesterol, HDL cholesterol, LDL cholesterol and triglyceride levels. The estimated glomerular filtration rate (eGFR) was calculated using the Modification of Diet in Renal Disease formula.

Transthoracic echocardiography was carried out using a Vivid S5 GE Healthcare system. Each patient underwent two-dimensional transthoracic echocardiography according to the recommendations of the European Association of Echocardiography [24]. Parasternal long axis view was used to view the proximal AA. The ascending aortic diameters were calculated between the inner edges of the aortic lumen perpendicular to the long axis 2 cm above the sinotubular junction at the end of the diastole in views showing the largest aortic diameter [16]. The parasternal long axis view was performed to measure maximal EAT thickness. EAT was defined as echo-free space between the outer wall of the myocardium and the visceral layer of the pericardium [14]. PAT was defined as the hypoechoic space in front of EAT and on the external pericardium [14–25]. The values were measured in three cardiac cycles and were averaged.

### Statistical analysis

The SPSS 13.0 (SPSS Inc., an IBM Company, Chicago, USA) was used for statistical analyses. Data were tested for distribution using the Kolmogorov–Smirnov test. Categorical variables were introduced as percentages. Continuous variables were introduced as mean ± SD, and abnormally distributed variables are introduced as median (25–75 percentages). Normally distributed continuous variables were analysed with the 2-tailed Student’s *t* test, and not normally distributed variables were analysed with the Mann–Whitney *U* test. Categorical data were analysed using the Fisher’s exact test or chi square. Pearson and Spearman tests were used for correlation analysis. Since the study was nonrandomized, a logistic regression model with propensity scores was created in order to balance patient characteristics and perform propensity-matched analysis of the patients with and without AAD. Clinical determinants of the hypertensive patients with AAD were established using univariate and multivariable logistic regression analyses. Logistic regression analyses were performed for the multivariate analysis of independent variables, which were included if they were statistically significant in the univariate analyses. Receiver-operating characteristic (ROC) curve graphics were used to determine the cutoff value of EAT. A *p* value < 0.05 was accepted as statistically significant.

## Results

A total of 934 hypertensive patients were divided into the following two groups: patients with ascending aortic dilatation (AAD) (102 patients) and patients with normal ascending aorta (AA) (832 patients). Baseline characteristics and echocardiographic and biochemical parameters are summarised in Table 1.

**Table 1:** Baseline characteristics of patients with and without aortic dilatation

Characteristic	Aortic Dilatation (*n* = 102)	Normal Aorta (*n* = 832)	*P*-value
Age (years)	72.3 ± 11.6	61.7 ± 12.7	< 0.001
Female *n* (%)	67 (66)	446 (54)	0.021
Body mass index (kg/m^2^)	26.8 ± 4.5	30.7 ± 4.9	< 0.001
BSA (m^2^)	1.70 ± 0.19	1.90 ± 0.19	< 0.001
Height (m)	156 ± 8	164 ± 9	< 0.001
Weight (kg)	66 ± 13	83 ± 14	< 0.001
Coronary Artery disease *n* (%)	29 (29)	195 (23)	0.265
Diabetes mellitus *n* (%)	18 (18)	245 (29)	0.012
Dyslipidemia *n* (%)	33 (32)	272 (33)	0.945
*Echocardiographic parameters*			
Ejection fraction (%)	57.6 ± 7.4	59.4 ± 5.7	0.005
Epicardial adipose tissue (cm)	0.48 ± 0.15	0.44 ± 0.11	< 0.001
Paracardial adipose tissue (cm)	0.49 ± 0.18	0.49 ± 0.17	0.738
Ascending aortic diameter (mm)	38.5 ± 4.2	34.0 ± 3.6	< 0.001
LVEDD (mm)	48.6 ± 4.6	47.8 ± 4.0	0.092
Left atrium diameter (mm)	38.2 ± 5.9	36.4 ± 5.7	0.004
Right ventricular diameter (mm)	23.1 ± 2.5	23.2 ± 2.9	0.628
Left ventricular hypertrophy *n* (%)	44 (44)	280 (34)	0.042
E	80 ± 25	78 ± 24	0.461
A	89 ± 18	83 ± 18	0.003
E/A	0.9 ± 0.3	1.0 ± 0.3	0.057
e’	6.9 ± 1.7	7.3 ± 1.9	0.046
E/ e’	11.9 ± 3.3	11.3 ± 4.4	0.158
Mitral regurgitation			< 0.001
**Grade-1*n*(%)**	25 (25)	155 (19)	
Grade-2 *n* (%)	14 (14)	50 (6)	
**Grade-3*n*(%)**	4 (4)	9 (1)	
**Aortic regurgitation**			< 0.001
Grade-1 *n* (%)	30 (29)	129 (16)	
Grade-2 *n* (%)	20 (20)	47 (6)	
Grade-3 *n* (%)	4 (4)	3 (0)	
Tricuspid regurgitation			0.007
Grade-1 *n* (%)	42 (41)	342 (41)	
Grade-2 *n* (%)	15 (15)	68 (8)	
Grade-3 *n* (%)	10 (10)	38 (5)	
Aortic stenosis			< 0.001
Grade-1 *n* (%)	9 (9)	12 (1)	
Grade-2 *n* (%)	1 (1)	5 (1)	
Grade-3 *n* (%)	1 (1)	0 (0)	
*Medications*			
Calcium canal bloker *n* (%)	46 (45)	401 (48)	0.554
ACE -I/ARB *n* (%)	65 (64)	553 (67)	0.581
Beta-bloker *n* (%)	48 (47)	275 (33)	0.005
Doxazosine *n* (%)	2 (2)	31 (4)	0.362
Thiazide diuretic *n* (%)	46 (46)	415 (50)	0.450
Statin *n* (%)	24 (24)	151 (18)	0.189
Acetylsalicylic acid *n* (%)	23 (23)	192 (23)	0.905
*Biochemical parameters*			
Hemoglobin (g/dl)	12.7 ± 2.0	13.4 ± 1.7	< 0.001
WBC (/mm^3^)	6.9 ± 2.0	7.3 ± 2.1	0.085
Platelet (× 10^3^/μL)	232 ± 79	249 ± 74	0.032
eGFR (ml/min)	68.9 ± 22.0	78.1 ± 19.1	< 0.001
Glucose (mg/dl)	10.9.6 ± 24.7	125.7 ± 53.0	0.003
HbA1c (%)	6.2 ± 0.7	6.5 ± 1.3	0.033
Total Cholesterol (mg/dl)	201 ± 27	200 ± 29	0.876
LDL Cholesterol (mg/dl)	123 ± 32	118 ± 30	0.108
HDL Cholesterol (mg/dl)	48 ± 11	47 ± 10	0.324
Triglyceride (mg/dl)	132 (92–180)	167 (109–209)	0.004
AST (U/L)	19 (17–23)	22 (18–26)	0.002
ALT (U/L)	14 (12–19)	18 (14–25)	< 0.001

*BSA* body surface area, *AST* Aspartate transaminase, *ALT* Alanine transaminase, *WBC* White blood cell count, *LVEDD* Left ventricular end diastolic diameter, *eGFR* Estimated glomerular filtration rate. *ACE* Angiotensin-converting enzyme inhibitors, Angiotensin II receptor blockers, *BMI* body mass index

Patients with AAD were older than the patients with normal AA (72.3 ± 11.6 vs. 61.7 ± 12.7 years, *p* <  0.001), had more female gender (66% vs. 54%,*p* = 0.021) compared with patients with normal ascending aorta (AA). BMI and BSA were lower in patients with AAD than patients with normal AA (Table 1). EAT thickness (0.48 ± 0.15, *p* <  0.001) was significantly higher in the patients with AAD compared with the patients with normal AA. There was no difference between groups regarding PAT thickness (0.49 ± 0.18 vs 0.49 ± 0.17, *p* = 0.738). β-Blocker usage is higher in patients with AAD than patients with normal AA.

In biochemical analyses, eGFR was lower in patients with AAD than patients with normal AA (68.9 ± 22.0 vs 78.1 ± 19.1, *p* <  0.001).

After propensity score matching analysis (77 vs*.* 77), EAT and age were significantly higher in patients with AAD than those without AAD (0.49 ± 0.16 vs 0.42 ± 0.10, *P* = 0.002; 69.8 ± 11.4 vs 64.4 ± 11.3, *p* <  0.001, respectively). Characteristics of the population after matching are presented in Table 2. In the matched population, EAT was associated with AAD in multivariate logistic regression analysis [OR:1.461, %95CI (1.082–1.974), *p* = 0.013] (Table 3).

**Table 2:** Baseline characteristics of patients with and without aortic dilatation after matching.

Characteristic	Aortic Dilatation (*n* = 77)	Normal Aorta (*n* = 77)	*P*-value
Age (years)	69.8 ± 11.4	64.4 ± 11.3	< 0.001
Female *n* (%)	50 (65)	48 (62)	0.014
Body mass index (kg/m^2^)	27.8 ± 4.5	29.6 ± 5.1	0.018
BSA (m^2^)	1.74 ± 0.16	1.87 ± 0.25	< 0.001
Height (m)	158 ± 8	162 ± 11	0.021
Weight (kg)	70 ± 11	78 ± 18	0.001
Coronary Artery disease *n* (%)	25 (33)	13 (17)	0.025
Diabetes mellitus *n* (%)	15 (15)	15 (15)	1.000
Dyslipidemia *n* (%)	28 (36)	23 (30)	0.392
*Echocardiographic parameters*			
Ejection fraction (%)	58.0 ± 7.3	58.5 ± 6.2	0.679
Epicardial adipose tissue (cm)	0.49 ± 0.16	0.42 ± 0.10	0.002
Paracardial adipose tissue (cm)	0.49 ± 0.18	0.49 ± 0.17	0.941
Ascending aortic diameter (mm)	39.0 ± 3.8	29.3 ± 3.1	< 0.001
LVEDD (mm)	48.8 ± 4.5	46.8 ± 4.3	0.005
Left atrium diameter (mm)	38.3 ± 6.1	35.0 ± 6.9	0.002
Right ventricular diameter (mm)	23.1 ± 2.5	22.7 ± 3.4	0.416
Left ventricular hypertrophy *n* (%)	32 (42)	23 (30)	0.130
E	80 ± 25	78 ± 21	0.610
A	87 ± 18	86 ± 16	0.639
E/A	0.9 ± 0.3	0.9 ± 0.2	0.478
e’	7.1 ± 1.6	7.5 ± 2.0	0.234
E/ e’	11.7 ± 3.3	11.1 ± 3.6	0.344
Mitral regurgitation			0.189
**Grade-1*n*(%)**	18 (23)	19 (25)	
Grade-2 *n* (%)	11 (14)	5 (7)	
**Grade-3*n*(%)**	2 (0)	0 (0)	
**Aortic regurgitation**			0.710
Grade-1 *n* (%)	21 (27)	17 (22)	
Grade-2 *n* (%)	11 (14)	15 (20)	
Grade-3 *n* (%)	2 (3)	1 (1)	
Tricuspid regurgitation			0.595
Grade-1 *n* (%)	32 (42)	35 (46)	
Grade-2 *n* (%)	10 (13)	5 (7)	
Grade-3 *n* (%)	6 (8)	7 (9)	
Aortic stenosis			0.565
Grade-1 *n* (%)	4 (5)	3 (4)	
Grade-2 *n* (%)	0 (0)	(1)	
Grade-3 *n* (%)	0 (0)	0 (0)	
*Medications*			
Calcium canal bloker *n* (%)	35 (46)	35 (46)	1.000
ACE -I/ARB *n* (%)	50 (65)	47 (61)	0.617
Beta-bloker *n* (%)	31 (40)	24 (31)	0.239
Doxazosine *n* (%)	2 (3)	1 (1)	0.560
Thiazide diuretic *n* (%)	37 (48)	34 (44)	0.628
Statin *n* (%)	22 (29)	10 (13)	0.017
Acetylsalicylic acid *n* (%)	21 (27)	11 (14)	0.047
*Biochemical parameters*			
Hemoglobin (g/dl)	12.9 ± 1.8	13.0 ± 1.8	0.671
WBC (/mm^3^)	7.2 ± 2.0	7.1 ± 2.1	0.639
Platelet (×10^3^/μL)	243 ± 74	250 ± 83	0.570
eGFR (ml/min)	72.2 ± 21.2	77.4 ± 16.5	0.093
Glucose (mg/dl)	112.3 ± 26.8	108.1 ± 25.1	0.320
HbA1c (%)	6.3 ± 0.7	6.2 ± 0.9	0.474
Total Cholesterol (mg/dl)	201 ± 31	201 ± 25	0.965
LDL Cholesterol (mg/dl)	120 ± 33	120 ± 32	0.836
HDL Cholesterol (mg/dl)	48 ± 12	47 ± 10	0.774
Triglyceride (mg/dl)	134 (91–180)	140 (107–181)	0.994
AST (U/L)	19 (17–22)	22 (19–26)	0.231
ALT (U/L)	15 (12–18)	17 (13–24)	0.459

*BSA* body surface area, *AST* Aspartate transaminase, *ALT* Alanine transaminase, *WBC* White blood cell count, *LVEDD* Left ventricular end diastolic diameter, *eGFR* Estimated glomerular filtration rate. *ACE* Angiotensin-converting enzyme inhibitors, Angiotensin II receptor blockers, *BMI* body mass index

**Table 3:** Univariate and multivariate analysis of ascending aort dilatation

Variables	Univariate OR (95% CI)	*P*-value	Multivariate OR(95% CI)	*P*-value
Age (years)	1.030 (1.006–1.056)	0.016		
Coronary artery disease	1.548 (1.155–2.074)	0.027		
Female	1.119 (0.580–2.158)	0.738		
Statin	2.680 (1.171–6.135)	0.020		
ASA	2.250 (0.999–5.067)	0.050		
**eGFR (mL/min)**	0.985 (0.969–1.003)	0.014		
EAT thickness (mm)	1.548 (1.155–2.074)	0.003	1461 (1.082–1.974)	0.013

*eGFR* Estimated glomerular filtration rate, *ASA* acetylsalicylic asid, *EAT* Epicardial adipose tissue

**Fig. 1:**
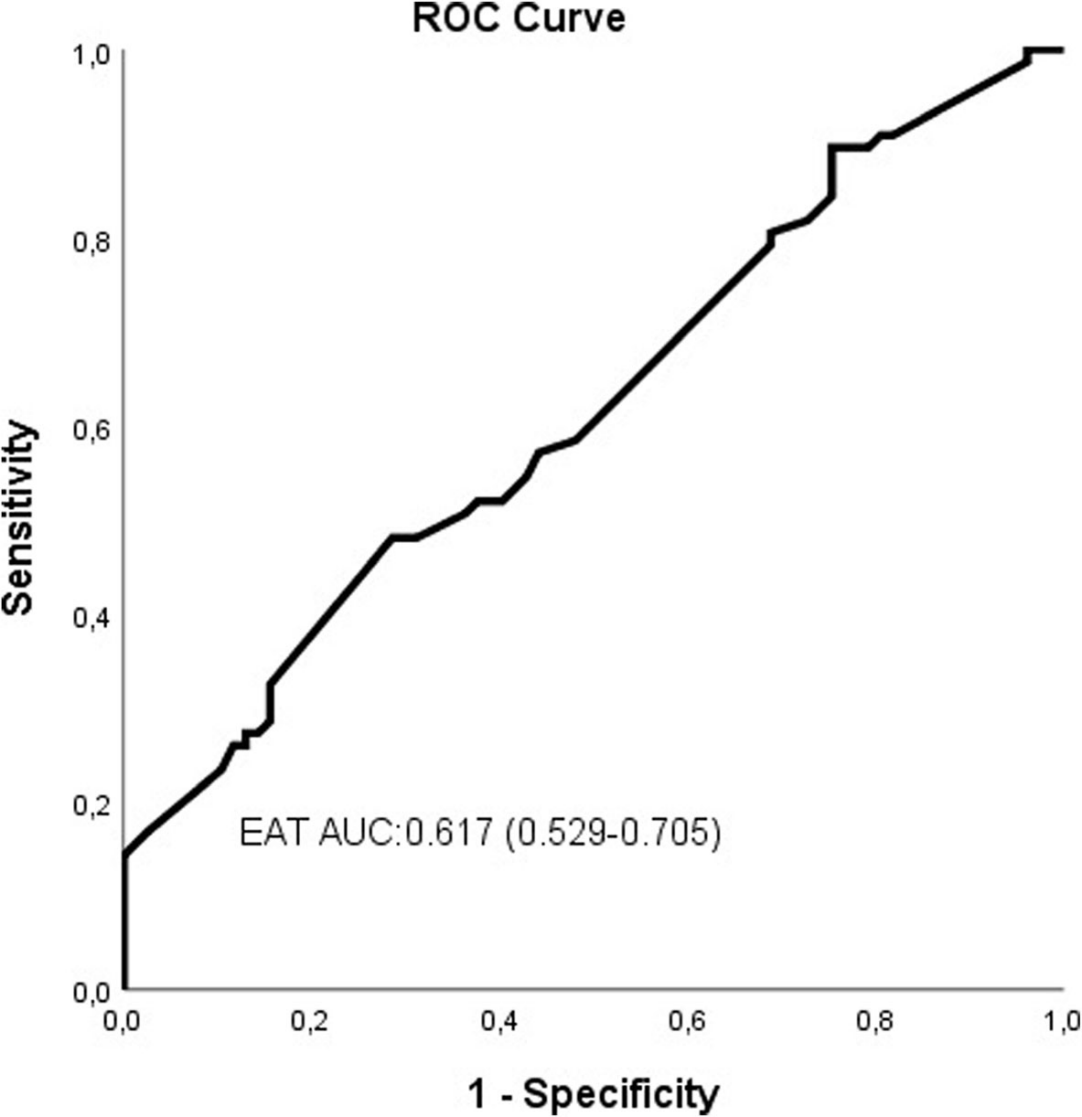
Receiver operating characteristic (ROC) curves for EAT in predicting of AAD

ROC curve analysis was performed to predict AAD in the matched population (Fig. 1). EAT > 0.45 cm had 51.9% sensitivity and 62.3% specificity [AUC = 0.617, *P* = 0.012, 95% CI (0.529–0.707)].

## Discussion

In this study, we found that EAT are independent predictor for AAD in hypertensive patients.

The normal diameter of the AA depends on the patient’s age, sex and body size [26]. Similarly to our study, Bon et al. showed that higher age was associated with larger descending aortic diameters. The causes of aortic aneurysms differ according to the location of the aorta. In younger patients, thoracic aortic aneurysms with genetic reasons generally include the AA and aortic root. These reasons include connective tissue disorders, such as Ehlers-Danlos Syndrome, Marfan Syndrome, Loeys–Dietz Syndrome, Familial Thoracic Aortic Aneurysm Syndrome, Turner Syndrome and bicuspid aortic valve. Also, cystic medial degeneration can be seen in thoracic aortic aneurysms that are not due to a connective tissue disorder but have a genetic connection. Other reasons of thoracic aortic aneurysms are aortitis (syphilis is the most common cause) or inflammation of the aorta. A total of 39% of the variance in descending aortic diameter and 21% of the variance in ascending aortic diameter were explained by sex, age, traditional cardiovascular risk factors and anthropometrics [27]. Consequently, only a small part of the causes of ascending aortic aneurysm could be explained by conventional risk factors. The influence of these cardiovascular risk factors seems of limited importance in elucidating the overall reasons for aortic dilatation.

EAT may help fill this gap. There are two possible mechanisms for the association between EAT and AAD. First, EAT has a paracrine and systemic endocrine role. It can secrete several active molecules, such as tumour necrosis factor, resistin, adiponectin and IL-6 [28, 29]. Second, EAT is one of the parts of the visceral adipose tissue, and, therefore, it is associated with CAD, cardiovascular risk factors, hypertension and metabolic syndrome [30–32]. These specifications of EAT may be a possible causative role of AAD.

In the literature, PAT and EAT have been used interchangeably, because they were thought to be identical adipose depots. However, paracardial and epicardial adipose tissues are clearly different embriologically, physiologically, anatomically and clinically. Despite this, the literature frequently contradictorily discriminates between the two adipose tissues [33]. EAT is the fat depot placed between the myocardium and visceral pericardium, whereas PAT is the fat depot outside the pericardium and on the external surface of the parietal pericardium as defined by imaging studies and autopsies [14–34]. So, EAT is the adipose tissue depot immediately adjacent to the myocardium, whereas PAT is the outer adipose tissue of the heart.

EAT originates from the splanchnopleuric mesoderm, whereas PAT originates from the thoracic primitive mesenchyme. EAT is supplied by the coronary arteries, whereas PAT is supplied by the noncoronary system. Also, no any fascia separates the EAT and coronary arteries; therefore, EAT and myocardium share the same circulation. Because of this link, EFT is metabolically an active tissue and the source of cytokines and interacts directly through vasocrine and paracrine mechanisms to the myocardium, coronary and systemic circulation [34]. This is not true for PAT. PAT does not directly interact with the myocardium and may only contact indirectly through acts like ectopic visceral adipose tissue. As a result, pericardial and EATs are clinically different [35].

CAD is correlated with AAD in univariate regression analysis, but this correlation was not determined in multivariate regression analysis after matching. Atherosclerosis can lead to aortic dilatation, especially in aortic arch and descending aorta. Unlike dilatation of the descending aorta, the role of atherosclerosis in the aetiology of AAD is controversial, and AAD is not generally due to atherosclerosis. Most aetiology of the AADs are classified as idiopathic [36, 37].

Interestingly, the presence of DM was related to a smaller ascending and descending aortic diameter in the literature [38]. This phenomenon might be caused by high glucose levels associated with DM which inhibits intermediate-phase secretion of the matrix metalloproteinases. Similarly, protective effect of DM has already been shown in the abdominal aorta [39, 40]. However, this phenomenon may be more remarkable in abdominal aorta than AA. We did not find any relationship between DM and AAD.

It has been shown that there was a relationship between EAT density and arterial inflammation and cardiovascular risk prediction. This adipose tissue-arterial wall association may play a role in the formation of AAD [41].

The findings of this study may support the hypothesis that increased EAT, but not PAT, may have a critical role in AAD.

## Conclusion

Only a little part of the aetiology of AAD was explained by age, sex, traditional cardiovascular risk factors and anthropometrics. EAT may have a key role in AAD. Based on our findings, increased EAT, and BMI were independent predictors for AAD in hypertensive patients. Increased EAT may be effective in progressing aortic dilatation due to local or systemic effects. However, this relationship was not observed in PAT. We believe that further studies are needed to explain the role of different types of adipose tissue in the pathogenesis of AAD. Adipose tissue-focused diagnosis and treatments can provide new insight to the patients with AAD.

### Study limitations

This was primarily a cross-sectional study retrospective in nature. MRI and computed tomography are frequently used in the evaluation of adipose tissue. Although MRI is the modality of choice for water/fat separation, CT is considered to gold standard for volumetric adipose tissue analyses. However, we used echocardiography to measure EAT. Also, there are some limitations to the measurement of EAT and PAT by echocardiography. Echocardiographic imaging only allows for a rough estimation of the adipose tissue [42, 43]. The restricted acoustic window limits the imaging of total adipose tissue volume and determining regional differences in adipose tissue distribution. Nevertheless, there is a close correlation between MRI and echocardiography imaging of EAT [44, 45]. In our study, predictive value of EAT for AAD was modest. The cutt-off values of epicardial fat for AAD prediction are not well defined in literature. Whether or not echocardiographic epicardial fat thickness may really have the diagnostic properties to serve as an indicator of AAD should be analyzed in large, randomized studies.

## Data Availability

The datasets used and analyzed during the current study are available from the corresponding author on reasonable request.

## Abbreviations

EAT: Epicardial adipose tissue
AAD: Ascending aortic dilatation
BMI: Body mass index
PAT: Paracardial adipose tissue
BSA: Body surface area
CAD: Coronary artery disease
DM: Diabetes mellitus
AA: Ascending aorta
EF: Ejection fraction
LVEDD: Left ventricle end diastolic diameter
LA: Left atrium
Hb: Haemoglobin
Htc: Haematocrit
AST: Aspartate transaminase
ALT: Alanine transaminase
eGFR: Estimated glomerular filtration rate
